# Epigenetic Control of *SPI1* Gene by CTCF and ISWI ATPase SMARCA5

**DOI:** 10.1371/journal.pone.0087448

**Published:** 2014-02-03

**Authors:** Martina Dluhosova, Nikola Curik, Jarmila Vargova, Anna Jonasova, Tomas Zikmund, Tomas Stopka

**Affiliations:** 1 Department of Pathophysiology, First Faculty of Medicine, Charles University in Prague, Prague, Czech Republic; 2 Department of Medicine - Hematology, First Faculty of Medicine, Charles University in Prague, Prague, Czech Republic; Florida State University, United States of America

## Abstract

CCCTC-binding factor (CTCF) can both activate as well as inhibit transcription by forming chromatin loops between regulatory regions and promoters. In this regard, Ctcf binding on non-methylated DNA and its interaction with the Cohesin complex results in differential regulation of the *H19*/*Igf2* locus. Similarly, a role for CTCF has been established in normal hematopoietic development; however its involvement in leukemia remains elusive. Here, we show that Ctcf binds to the imprinting control region of *H19*/*Igf2* in AML blasts. We also demonstrate that Smarca5, which also associates with the Cohesin complex, facilitates Ctcf binding to its target sites on DNA. Furthermore, Smarca5 supports Ctcf functionally and is needed for enhancer-blocking effect at ICR. We next asked whether CTCF and SMARCA5 control the expression of key hematopoiesis regulators. In normally differentiating myeloid cells both CTCF and SMARCA5 together with members of the Cohesin complex are recruited to the *SPI1* gene, a key hematopoiesis regulator and leukemia suppressor. Due to DNA methylation, CTCF binding to the *SPI1* gene is blocked in AML blasts. Upon AZA-mediated DNA demethylation of human AML blasts, CTCF and SMARCA5 are recruited to the −14.4 Enhancer of *SPI1* gene and block its expression. Our data provide new insight into complex *SPI1* gene regulation now involving additional key epigenetic factors, CTCF and SMARCA5 that control PU.1 expression at the −14.4 Enhancer.

## Introduction

Control of tumor suppressor gene expression requires cooperation of epigenetic factors and represents crucial yet not fully understood events in leukemogenesis. Ctcf is a key epigenetic regulator and zinc-finger transcription factor, whose binding to DNA can be prevented by DNA methylation [1]. Ctcf regulates the well-established imprinted *H19*/*Igf2* locus by blocking communication between the *Igf2* promoter and its enhancer leading to increased transcription of *H19* on the maternal allele. Ctcf association within a cohesin complex at the *H19*/*Igf2* locus enables long-range chromatin interactions [2] and efficient enhancer blocking [3] on the unmethylated imprinting control region (ICR) of the maternal allele [4]. Several reports indicated Ctcf involvement in hematopoiesis [5] [6] and leukemogenesis [7]. However, the mechanisms by which Ctcf regulates these processes are not well understood.

Smarca5 (Snf2h), is an epigenetic chromatin-remodeling factor that assembles and slides nucleosomes along the DNA fiber. Smarca5 together with a bromodomain-containing protein WCRF180 has been demonstrated to load the Cohesin complex onto DNA [8]. Expression patterns of Ctcf and Smarca5 (together with the Cohesin complex) overlap in hematopoietic progenitor cells (www.biogps.org). Moreover, Smarca5 is required for proliferation and/or differentiation of immature hematopoietic progenitors with extensive cytokine-induced proliferative capacity [9] and is upregulated in acute myeloid leukemia (AML) blasts [10].

The transcription factor PU.1 (*SPI1*, *Sfpi1*) is a key regulator of hematopoiesis. The expression levels of PU.1 are precisely controlled by several myeloid transcription factors, including PU.1 itself [11], through interactions at an upstream regulatory element (URE) and the promoter [12]. The URE directs >80% of PU.1 expression (dependent on cell type) and its deletion in mouse or inactivation by a provirus leads to AML [13]. Additional enhancers between the URE and the *SPI1* promoter have been suggested to regulate long-range interactions at the *SPI1* locus [14]. Our recent work demonstrated that the URE is methylated in a subset of Myelodysplastic syndrome (MDS) and AML patients. Moreover, demethylation by 5-azacitidine (AZA) treatment increased PU.1 expression and induced myeloid differentiation [15]. This suggests that the *SPI1* gene in AML may become epigenetically dysregulated due to defective binding of factors that recognize unmethylated DNA.

We herein show that CTCF together with SMARCA5 and members of the Cohesin complex associate with the *SPI1* gene in normal myeloid cells, but this interaction is disrupted in AML blasts. Upon DNA demethylation in leukemia cells, the recruitment of CTCF and its binding partners is partially restored. Moreover, CTCF binding is newly established at the −14.4 Enhancer leading to a block in PU.1 expression. Thus in leukemia cells, AZA exposes newly hypomethylated sites to CTCF that together with SMARCA5 bind and control PU.1 expression.

## Materials and Methods

### Cell Lines

MEL cells were cultured as described elsewhere [16] and transfected (DMRIE-C, LifeTech.) with 25 nM siRNA-CTCF or non-silencing control siRNA (L-044693-01, Dharmacon). Stable transgenic Smarca5-shRNA cells were kindly obtained from Prof. Arthur. I. Skoultchi Laboratory. shSmarca5 was activated with 1 µg/mL of doxycycline. 2×10^6^ OCI-M2 cells [15] (DSMZ) were transfected (Amaxa) with 20 pmol siRNA-hSMARCA5 (s16082, Ambion, control: #1-AM4611), or with 0,5 µg pCTCF (Origene) or pBSK+ plasmid negative control. OCI-M2 were treated with 5 µM AZA (3-doses in 72 hrs) in presence of granulocyte colony-stimulating factor (G-CSF) (50 ng/ml) [15].

### Primary Samples

Written donor’s informed consent (based on 1964 Declaration of Helsinki) was obtained. *The Ethics Committee of the General Faculty Hospital and First Medical Faculty, Charles University* approved the project. CD34+ cells were magnetically separated and their quality assessed by flow cytometry (purity >95%). Bone marrow samples from two AML patients with dysplastic features and two advanced high-risk MDS were utilized (see Table S1). Normal CD34+ cells were magnetically separated from normal donor peripheral blood. Mixed myeloid cells were unfractionated peripheral blood mononuclear myeloid cells derived from G-CSF-mobilized donors in complete remission of lymphoma. Mixed myeloid cells exceeded 95% (as determined by differential blood count) of granulocytes, monocytes and their precursors. Depletion of erythrocytes was done by ammonium chloride.

### RNA and Protein Assays

TaqMan RT-PCR (384well-7900HT instrument, LifeTech.): cycling 40x 10 s/95°C, 20 s/60°C, 30 s/72°C. Analyses involved standard curve equation and linear CT transformation using δδCT equation (primers: Table S2).

Western blot lysates (RIPA) were gently (3×1 s) sonicated on Branson Sonic Dismembrator (model 500) with a micro-tip. SDS-PAGE was run, dry-blotted and incubated with antibodies: anti-Smarca5 (cat.# 07–624, Upstate, 1∶500), anti-Ctcf (cat.# 07–729, Upstate, 1∶5000), anti-PU.1 (sc-352, Santa Cruz, 1∶600), secondary-HRP-conjugated anti-rabbit (cat.# 80919, Jackson ImmunoResearch, 1∶4000), HRP-conjugated anti-actin (I-19), sc-1616; Santa Cruz).

Nuclear immunostaining was done with anti-Smarca5 (cat.# MAB3644, Chemicon, 1∶200), anti-Ctcf (cat.# sc-28198, Santa Cruz, 1∶500), secondary antibodies (cat.# A-11020, LifeTech., 1∶300 or cat.# A- 11070, 1∶300). Heterochromatin staining: Vectashield Mounting Medium containing 4′,6-diamidino-2-phenylindole (DAPI). Confocal laser scanning microscopy: Leica TCS SP2 with AOBS system.

### Enhancer-blocking Assay

The reporter vectors pIHLE, pIHLME and pIHLIE are based on the pGL3 luciferase reporter plasmid (Promega). The vectors were generated and kindly provided by Prof. Mitsuyoshi Nakao from Kumamoto, Japan. The reporter assay has been described previously [17] [18]. For knockdown experiment the HeLa cells were co-transfected with siRNA targeting CTCF, SMARCA5 or non-specific siRNA together with reporter vectors and Renilla luciferase control plasmid using lipofectamine (Invitrogen). The cells were lysed 96 hrs after transfection and analyzed using Dual Luciferase Assay and luminometer Glomax (all Promega). Firefly activity was normalized on Renilla luciferase activity.

### DNA Methylation, Co-IP and ChIP

Genomic DNA was treated using bisulfite, amplified (primers in Table S2), and subcloned. DNA from clones (>10 clones/amplicon) was sequenced as described elsewhere [15].

For co-IP, 6×10^6^ MEL or K562 cells were lysed (RIPA) and gently sonicated. 100 µg of precleared protein extract was incubated 3 hrs/4°C with anti-Ctcf (cat#07–729) or anti-Smarca5 (cat#07–624, Upstate) and next with proteinA/proteinG overnight. Control antibody: IgG, cat.# NI01, Calbiochem, 5∶100). Immunoprecipitates (IP) were washed with set of buffers. IPbuffer (0.02% SDS/2%Trion X-100/4 mM EDTA/40 mM Tris-HCl (pH = 8)/300 mM NaCl), WashI (0.1%SDS/1%Triton X-10/2 mM EDTA (pH = 8)/20 mM Tris-HCl (pH = 8)/50 mM NaCl), WashII (0.1% SDS/1% Triton X-10/1% EDTA (pH = 8)/20 mM Tris-HCl (pH = 8)/500 mM NaCl). IPs were resolved on SDS/PAGE, blotted, and immune-detected.

Chromatin immunoprecipitation (ChIP) [19] lysates were controlled for DNA purity&quantity by Nanodrop ND-1000. Antibodies: Smarca5/Snf2h (cat.#ab3749, Abcam, 3 µg/IP), Ctcf (cat.#ab10571, Abcam, 2 µg/IP), RAD21 (cat.#ab992, Abcam, 2 µg/IP), and SMC1 (cat.# ab9262, Abcam, 2 µg/IP). Control IgG: cat.#NI01, Calbiochem, 5∶100.

## Results

### 1. Smarca5 Regulates Ctcf Recruitment onto the ICR Region

**Figure 1:**
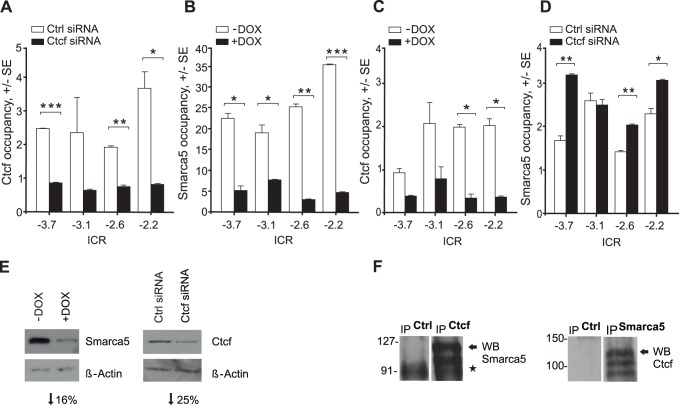
Ctcf and Smarca5 interact in AML cells. **A** Ctcf occupancy at ICR. ChIP of Ctcf-siRNA-treated MEL cells (Ctcf siRNA, black bars) or non-specific siRNA (Ctrl siRNA, white) at 72 hrs. Y-axis: specific IP DNA fragment enrichment over control IP (standard error, SE). X-axis: ICR amplicons (relative to H19-TSS). Asterisks: p-values (t test, 0.05–0.005). B Smarca5 occupancy at ICR. MEL-shSmarca5 treated 48 hrs with doxycycline (+DOX, black bars) or untreated (-DOX, white). C Ctcf occupancy is decreased at the ICR upon Smarca5 knockdown. Occupancy of Ctcf (lysates from 1B) determined by ChIP. D Smarca5 at ICR upon Ctcf knockdown. Occupancy of Smarca5 (lysates from 1A) by ChIP. E Knock-down of Smarca5 and Ctcf. Protein lysates from samples 1A and 1B were analyzed by Immunoblotting. Migration of Ctcf, Smarca5, and β-actin bands are indicated. Level of downregulation (bellow blots) was determined by densitometry. F Co-IP of Smarca5 and Ctcf in MEL cells. Antibodies for IP and detection are indicated; asterisk indicates nonspecific signal.

The role of Ctcf was previously well documented in mouse epigenetics [1] and it was suggested to regulate differentiation of leukemic cells [5]. To test whether in AML blasts Ctcf binds at the ICR and regulates the expression of the *H19* and *Igf2* genes, we first used mouse erythroleukemia (MEL) cells. MEL cells express the H19 mRNA and the Igf2 mRNA, albeit at approximately 1000-fold lower level (Figure S1), indicating that Ctcf may associate with the ICR of *H19*/*Igf2*. Indeed, Ctcf was detected at the ICR by ChIP at four neighboring amplicons (−3.7, −3.1, −2.6, −2.2 kb relative to H19 TSS) in MEL cells (Figure 1A, white bars). Detection of Ctcf at the ICR was specific as indicated by its depletion following Ctcf knockdown (Figure 1A, black bars). Efficiency of the Ctcf knockdown was confirmed using Western blots (Figure 1E right panel).

Ctcf has been proposed to require epigenetic remodeling and that the ICR is regulated by interplay between Ctcf and putative nucleosome-remodeling factors and DNA methylation machinery within the ICR [20] [21]. As such, we tested the involvement of the ISWI nucleosome assembly and sliding ATPase, Smarca5 at the ICR. We used MEL cells with a stably integrated doxycycline (Dox)-dependent Smarca5-shRNA construct. Using ChIP we established Smarca5 occupancy at the ICR coincidently with Ctcf (Figure 1B white bars). Efficiency of Smarca5-shRNA was confirmed on mRNA (Figure S2), protein (Figure 1E left) and occupancy (Figure 1B) levels. In addition, using different antibody we again detected Smarca5 occupancy at the ICR (Figure 1D, white bars). Outside ICR we tested number of loci where occupancy of either Smarca5 of Ctcf was not detected (data not show).

As Ctcf and Smarca5 coincidently occupied the ICR it suggested that Ctcf may recruit Smarca5 onto the DNA. Another possibility was that Smarca5 recruited Ctcf or alternatively, Ctcf is recruited alone but interacts with Smarca5 that was recruited to the DNA by other mechanisms. To elucidate these possibilities the ChIP was performed on the ICR locus upon Smarca5 knockdown. Figure 1C shows that downregulation of Smarca5 resulted in depletion of the Ctcf occupancy. We next asked whether Smarca5 occupancy is in turn dependent on Ctcf. Surprisingly, upon siRNA-mediated CTCF knockdown we observed that occupancy of Smarca5 was stable (at one amplicon) or increased upon siRNA-mediated depletion of Ctcf (Figure 1D). Thus it is likely that Ctcf recruitment to its DNA binding sites at the ICR is dependent on the presence of Smarca5.

We next tested whether there is an interaction between Ctcf and Smarca5 by a co-immunoprecipitation assay. Indeed, antibody to Smarca5 was able to precipitate Ctcf protein from total lysates as revealed by immunoblotting using antibody to Ctcf (Figure 1F right). Conversely, the antibody to Ctcf immunoprecipitated the Smarca5 protein that was further revealed on the Smarca5 immunoblot (Figure 1F left). According to antibody datasheets, the signal for both Ctcf and Smarca5 consists of one dominant band and also inconsistently appearing additional less frequent band-signals upon high exposure. To establish Ctcf-Smarca5 interaction in erythroleukemias the co-IP was reproduced also in additional AML-erythroleukemia cell line (human K562 cells, Figure S3). To test Ctcf-Smarca5 interaction on cellular level the confocal microscopy of MEL nuclei was used and it revealed partial overlap of intensity profiles (Figure S4). To summarize this part, using ChIP, co-IP, and additional approaches in transformed blasts with the use of knockdown experiments: the Ctcf and Smarca5 interaction in AML-M6 was revealed.

### 2. SMARCA5 Functionally Cooperates with CTCF

**Figure 2:**
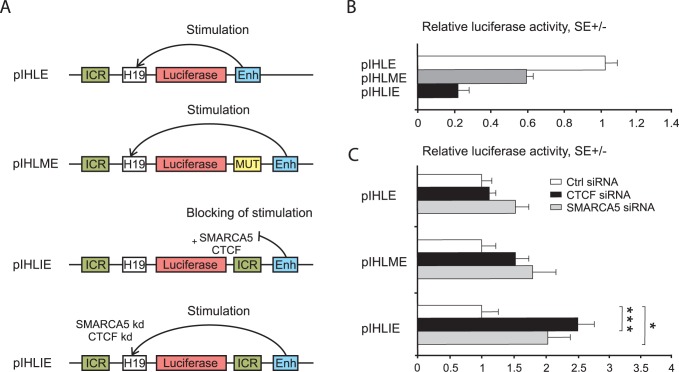
SMARCA5 cooperates with CTCF on enhancer-blocking activity of *H19* promoter. **A:** schematic representation of the reporter constructs pIHLE (without ICR), pIHLME (with mutated CTCF bindng site at the ICR) and pIHLIE (with ICR that can bind CTCF). ICR, insulator of *H19* gene; H19, promoter of *H19* gene; luciferase, luciferase gene; Enh, SV40 enhancer; MUT, insulator with mutated CTCF binding sites; kd, protein knock-down. **B:** Luciferase activity of reporter constructs in HeLa cells. Luciferase acitivity was normalized on Renilla, the pIHLE was set equal to 1. **C:** HeLa cells depleted for either CTCF or SMARCA5 (using siRNAs) were analyzed for luciferase activity of reporters. Luciferase activity was normalized on non-specific siRNA (Ctrl siRNA) that was set equal to 1, error bars indicate standard errors (SE), t-test (p<0.05 indicated by star).

To test whether SMARCA5 is capable of regulating CTCF-mediated transcriptional outcome we utilized reporter assays with the vectors pIHLE, pIHLME and pIHLIE, as described previously [17] [18]. Briefly, while pIHLE contains the H19 promoter and enhancer and its activity is not affected by CTCF, in the pIHLIE the ICR insulator is placed between the promoter and enhancer and upon CTCF binding it efficiently blocks transcriptional activity of the reporter. pIHLME is similar to pIHLIE with exception of CTCF-binding site mutation (Figure 2A). While transcriptional activity of pIHLIE is inhibited in presence of CTCF, the pIHLME activity is 3-fold higher (Figure 2B). To test requirement of SMARCA5 in the CTCF-mediated enhancer-blocking reporter system we inhibited SMARCA5 by siRNA and determined luciferase activity of the above-mentioned plasmids transiently transfected into the HeLa cells. Indeed, upon knockdown of either CTCF or SMARCA5 the reporter activity was significantly increased (Figure 2C). Therefore, SMARCA5 is an important epigenetic factor required for CTCF enhancer-blocking activity (see also Figure 2A, at the bottom).

### 3. Smarca5 Regulates Transcriptional Outcome of Ctcf Target Genes

**Figure 3:**
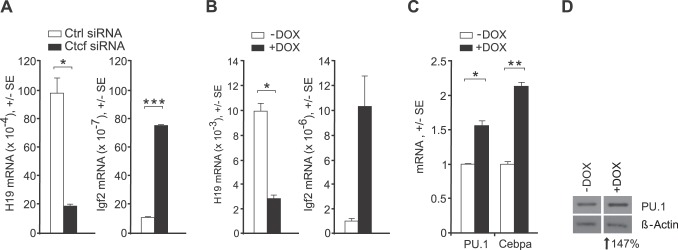
Smarca5 regulates Ctcf target genes. **A:** H19 and Igf2 mRNA expression upon Ctcf knockdown (Ctcf siRNA) or Ctrl siRNA. **B:** Smarca5 knockdown (+DOX) compared to untreatment (-DOX). RT-PCR analyses (A&B) were done at 72hrs. Y-axis: specific mRNAs relative to Hprt1 levels. **C:** mRNA levels of PU.1 and Cebpa at 96 hrs (4 days) upon Smarca5 knockdown. Y-axis: specific mRNA relative to average of Hprt1 and Gapdh was normalized on negative control (non-specific siRNA). Error bars: the standard errors (SE). Asterisks: p-values (t-test, 0.05–0.005). D: PU.1 and β-actin expression determined by Immunoblotting at 144 hrs (6 days) upon Smarca5 knockdown. Level of downregulation (bellow blots) was determined by densitometry.

We next asked whether manipulation of the Ctcf and Smarca5 would result in changes of *Igf2/H19* gene expression. MEL cells depleted of Smarca5 or Ctcf were analyzed by RT-PCR for mRNA expression. As expected [1], knockdown of Ctcf decreased levels of H19 (5-fold) and increased levels of Igf2 expression (7.5-fold) (Figure 3A). Similarly, knockdown of Smarca5 resulted in similar effects, downregulation of H19 (3-fold) and upregulation of Igf2 (10-fold) (Figure 3B). Thus, downregulation of Ctcf or Smarca5 result in similar transcriptional outcomes of the Ctcf target genes. This observation complements ChIP and reporter data and indicates that Smarca5 enables Ctcf to bind to ICR and stimulate H19 expression while inhibiting Igf2 transcription.

We next asked whether Ctcf/Smarca5 regulates genes directly involved in hematopoiesis and leukemogenesis. *Sfpi1* is a predicted CTCF-target gene and at the same time a well-known transcriptional master regulator of myelopoiesis [22]. It is known that Smarca5 is overexpressed in leukemic cells including MEL cells [10]. PU.1 is also expressed in MEL cells however its level is not downregulated therefore inhibiting the erythroid program. Coincidently such PU.1 level is not sufficient for inducing and completing the myeloid program whereas upon its further increase the MEL cells are capable of propagating the myeloid program [19]. Therefore, we used Smarca5 knockdown approach and tested effect of Smarca5 downregulation on expression of PU.1 and its target *Cebpa*. Indeed, shRNA-mediated downregulation of Smarca5 in MEL cells resulted in significant upregulation of PU.1 (and Cebpa) on mRNA (Figure 3C) and protein (Figure 3D) levels indicating that Smarca5 may regulate PU.1 levels in blasts.

### 4. CTCF/SMARCA5 are Recruited at Additional CTCF Target: *SPI1* Gene

**Figure 4:**
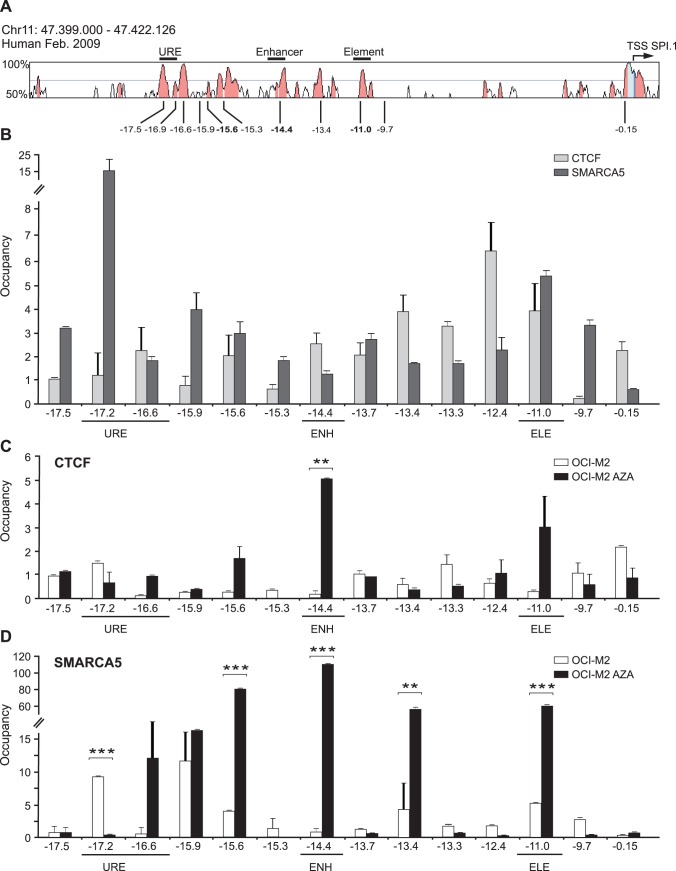
CTCF/SMARCA5 are recruited to *SPI1* locus in myeloid cells and upon AZA treatment in AML. **A:** Sequence conservation of human *SPI1* locus (VISTA) generated by aligning with murine DNA. Regulatory regions and positions of ChIP amplicons are numbered in respect to human *SPI1* TSS. **B:** ChIP of CTCF and SMARCA5 in mixed myeloid cells. **C:** ChIP of CTCF and **D:** SMARCA5 in OCI-M2 without (OCI-M2) or with AZA (OCI-M2 AZA) treatment. Y-axis: ChIP enrichment. X-axis: amplicons (distance relative to *SPI1* TSS). URE, Upstream Regulatory Element of *SPI1* gene; ENH, enhancer; ELE, element. Error bars: the standard errors (SE). Asterisks: p-values (t-test, 0.05–0.005).

As shown in Figure 4A, the human *SPI1* gene contains several highly conserved regions upstream from the TSS that contain previously described enhancers [14]. First, we asked whether CTCF and SMARCA5 occupancy are detectable in normal human myeloid cells. Using ChIP we focused on loci with more than 2-fold occupancy relative to control antibody. We identified diffuse occupancy of CTCF with peaks involving amplicons −16.6 (downstream URE), −14.4 (Enhancer), and −11 (Element) as well as in the neighboring amplicons: −15.6, −13.7, −13.4, −13.3, −12.4 and also close to the promoter at −0.15 (Figure 4B, grey bars). SMARCA5 occupancy was found also rather diffuse involving peaks from −17.5 to −15.3 (at URE and downstream URE) and at −11 (Element) and to lesser extent in the neighboring amplicons (Figure 4B, dark bars).

Next we tested CTCF and SMARCA5 occupancy at *SPI1* gene in human AML blasts of OCI-M2 cell line. *SPI1* gene was previously shown to be DNA-methylated in OCI-M2 and PU.1 expression is very low [15]. Not unexpectedly, CTCF was localized in just one of the 14 used amplicons near the *SPI1* promoter at −0.15 (Figure 4C, white bars). We observed distribution of SMARCA5 in AML blasts that was similar to mixed myeloid cells: from −17.2 to −15.6 (at URE and downstream URE) and at −11 (Element) (Figure 4D, white bars). Correspondingly to our knockdown experiments (Figure 1D) SMARCA5 occupancy was independent on CTCF occupancy. Strikingly, the predicted CTCF binding site at −14.4 Enhancer was occupied by neither CTCF nor SMARCA5 in AML blasts.

To test whether demethylation of *SPI1* gene would facilitate CTCF recruitment we used AZA treatment of OCI-M2 as we described recently [15]. Indeed demethylation of *SPI1* gene resulted in CTCF recruitment (Figure 4C, black bars). Strikingly, the CTCF recruitment induced by AZA was specifically localized at −14.4 (Enhancer). A trend towards CTCF occupancy is also seen at −15.6 and −11 (Element) that corresponds to CTCF occupancy in the mixed myeloid cells. AZA treatment facilitated SMARCA5 recruitment at the regulatory region of *SPI1* gene and overlapped with CTCF recruitment at −14.4 Enhancer (Figure 4D, black bars). In addition, the amplicons −15.6, −13.4, and −11 (Element) showed spread signal of SMARCA5. To conclude this part, CTCF and SMARCA5 co-occupy its newly validated target *SPI1* using ChIP assay at the −14.4 Enhancer upon AZA-mediated DNA demethylation.

### 5. Cohesin Members Co-occupy the *SPI1* Gene with CTCF and SMARCA5

**Figure 5:**
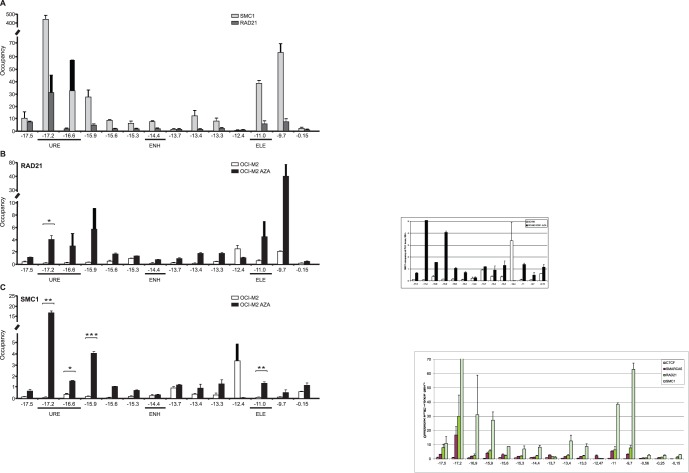
Binding of Cohesin complex members to *SPI1* locus. **A:** ChIP of RAD21 and SMC1 in mixed myeloid cells. **B:** ChIP of RAD21 and **C:** SMC1 in OCI-M2 without (OCI-M2) or with AZA (OCI-M2 AZA) treatment. Y-axis: ChIP enrichment. X-axis: amplicons (distance relative to *SPI1* TSS). URE, Upstream Regulatory Element of *SPI1* gene; ENH, enhancer; ELE, element. Error bars: the standard errors (SE). Asterisks: p-values (t-test, 0.05–0.005).

As stated in the introduction section, CTCF fractionates as part of the Cohesin complex. We asked whether this CTCF-Cohesin fraction is enriched at the *SPI1* gene. Using ChIP we tested occupancy of RAD21 and SMC1 using experimental conditions described in part 3 above. Occupancy at the *SPI1* regulatory regions by RAD21 and SMC1 was very similar. In normal human myeloid cells, RAD21 and SMC1 co-occupy the URE as indicated by amplicons −17.5 to −15.6. The second highest occupancy for RAD21 and SMC1 was observed at amplicons −11 (Element) and −9.7 (Figure 5A).

We next determined occupancy of RAD21 and SMC1 in AML blasts (OCI-M2) with or without previous AZA treatment. Indeed, lack of CTCF in OCI-M2 resulted in negligible occupancy of both RAD21 and SMC1 at the *SPI1* gene. Upon AZA treatment, RAD21 was recruited to −15.9, just upstream of CTCF and SMARCA5 binding at −15.6 (Figure 5B). Moreover, the signal spreads upstream towards the URE, together with SMARCA5. In addition, RAD21 was also recruited at −11 Element (where CTCF and SMARCA5 are also recruited) and at the −9.7 amplicon. SMC1 recruitment was observed only at one amplicon, at −17.2 at the URE (together with RAD21) (Figure 5C). Importantly, recruitment of CTCF and SMARCA5 at −14.4 Enhancer was not overlapped by RAD21 or SMC1 indicating that the Cohesin complex was not involved at this CTCF binding site.

### 6. CTCF/SMARCA5 Binding Site at *SPI1* Gene is Methylated in AML and Demethylated upon AZA

**Figure 6:**
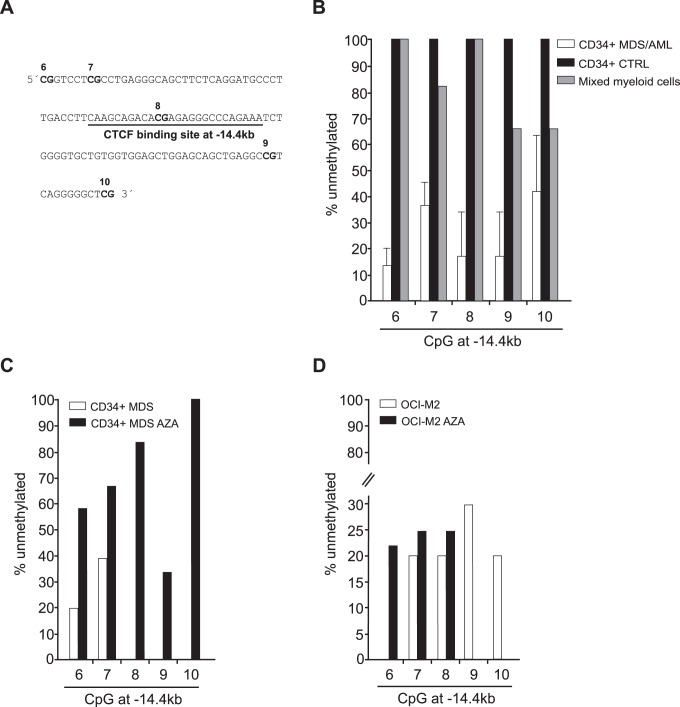
DNA methylation of CTCF binding site in *SPI1* locus. **A:** DNA sequence of the CTCF binding site at −14.4 kb Enhancer region within the *SPI1* locus (CGs are numbered on the top). **B:** % of DNA unmethylation identified by sequencing of bisulphite-treated DNA isolated from CD34+ cells of AML/MDS patients (N = 3, information in Table S1) and control CD34+ cell donors (N = 1) and mixed myeloid cells (N = 1) was performed at the region −14.4 kb of *SPI1* locus. The primer sequences are shown in Table S2. **C:** % of DNA unmethylation, data in CD34+ cells of MDS patient without AZA therapy (N = 1) and MDS patient treated by AZA (N = 1). **D:** % of DNA unmethylation in untreated OCI-M2 and AZA-treated OCI-M2. Y-axis: % of unmethylated CpGs; x-axis: number of CpG; error bars indicate standard errors.

To associate the DNA methylation status at the −14.4 Enhancer with CTCF occupancy, we used a sequencing approach of bisulfite-treated DNA. First, we mapped the CTCF binding site (Figure 6A) and determined the methylation status in AML CD34+ cells compared to normal CD34+ cells and mixed myeloid cells. Unlike its normal counterparts where the percentage of unmethylated DNA ranges from 70–100%, the MDS/AML progenitors showed a low level of unmethylated DNA ranging from 10 to 40% (Figure 6B). Interestingly, we compared effects of AZA treatment on the methylation status at the −14.4 Enhancer and observed that a patient treated with AZA contains more unmethylated CpGs compared to a patient who is not treated by demethylation therapy (Figure 6C). Effect of AZA at −14.4 on OCI-M2 is shown in Figure 6D. CTCF binding site is demethylated by 5% at CGs #7 and #8 and by 22% at CG #6. Compared to URE [15], where the effect of demethylation is very effective, in the −14.4 Enhancer the demethylation is less pronounced. At two additional CGs #9 and #10 downstream the CTCF binding AZA was unable to decrease DNA methylation. Complete set of DNA methylation data in OCI-M2 at −14.4 (where CTCF is enriched) and in the neighboring regions (−15.6 and −11, where CTCF enrichment shows trend towards significance) are shown in Figure S6A. In MDS patients treated with AZA the −14.4 Enhancer is a target of AZA-mediated demethylation (in CD34+ progenitors) within up to 6 neighboring CGs including those found in OCI-M2 (indicated by arrows, see Figure S6B). Interestingly, normal CD34+ cells display up to 10 demethylated CGs of the −14.4 Enhancer as compared to CD34+ cells from MDS/AML patients (Figure S6C). However, we didn’t detect these remarkably consistent DNA demethylation changes in other two regions (−15.6 and −11 Element) (Figure S6A–C).

### 7. Manipulation of either CTCF or SMARCA5 Affects Transcriptional Outcome of the *SPI1* Gene

**Figure 7:**
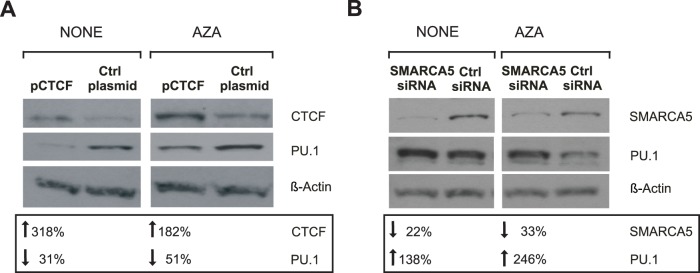
Perturbation experiments indicate effects of CTCF and SMARCA5 on PU.1 expression. **A:** Western blot of PU.1 in OCI-M2 after CTCF overexpression at 72 hrs. OCI-M2 cells were transfected with CTCF-encoding plasmid (pCTCF) or pBSK+ as negative control plasmid (Ctrl plasmid). AZA treatment (AZA) is indicated on the top of the lines. **B:** Western blot of PU.1 in OCI-M2 after downregulation of SMARCA5 at 72 hrs. OCI-M2 cells were transfected with siRNA to SMARCA5 and treated or untreated with AZA. Protein lysates were resolved by SDS/PAGE. Membrane with blotted proteins was immunostained with antibody to CTCF or SMARCA5, and PU.1. ß-actin was used as a control of sample loading. Level of down/up-regulation (small table) was determined by densitometry.

Recruitment of CTCF and SMARCA5 onto the *SPI1* gene in mixed myeloid cells as well as in AZA-treated OCI-M2 blasts indicated that CTCF and SMARCA5 might regulate the transcriptional outcome of *SPI1*. In order to address this possibility we decided to manipulate the levels of CTCF and SMARCA5 in human AML OCI-M2 blasts. Plasmid encoding the CTCF cDNA (pCTCF) or a SMARCA5 siRNA were transfected together with an additional plasmid encoding a green fluorescent protein (GFP) and immunoblots were performed on GFP positive cells. Perturbation experiments were done in OCI-M2 in absence of AZA as well as upon AZA that is known to stimulate expression PU.1 and its targets ([15] and Figure S5). We expected two possibilities: 1) CTCF/SMARCA5 stimulate PU.1 expression by binding to newly demethylated DNA and facilitating PU.1 transcription, possibly through URE. Alternatively, 2) CTCF and SMARCA5 might employ the enhancer blocking function upon binding to the −14.4 Enhancer and thereby prevent transcription from the promoter and inhibit PU.1 levels. Our data support the second possibility as the manipulation of CTCF levels (by overexpression from the transiently transfected plasmid), either in the absence of AZA or upon preincubation with AZA, resulted in inhibition of PU.1 expression (Figure 7A). Furthermore, downregulation of SMARCA5 levels (by transient siRNA-mediated knockdown) in OCI-M2, both in the absence of AZA or upon preincubation with AZA, resulted in upregulation of PU.1 expression and thereby complemented the CTCF perturbation experiment (Figure 7B). Thus, both perturbation experiments in OCI-M2 AML blasts favor the possibility of enhancer-blocking effects of CTCF/SMARCA5 at the −14.4 Enhancer. To summarize this part, we have provided evidence of direct link between recruitment of CTCF/SMARCA5 at newly demethylated DNA at *SPI1* regulatory regions and transcriptional outcome of *SPI1* gene in AML blasts.

## Discussion

Our work demonstrates an important role for a CTCF/SMARCA5 interaction in the regulation of gene expression in AML by showing cooperating activities of CTCF and SMARCA5 on the *SPI1* gene in AML blasts. First, we described the role of ATP-dependent chromatin remodeling factor Smarca5 during the process of Ctcf-ICR interaction. CTCF and SMARCA5 have been independently demonstrated to interact with cohesin [8] [18]. Cohesin and CTCF were found at the ICR where ChIP has demonstrated co-occupancy of CTCF and RAD21 and confirmed their cooperating effects on transcription [2]. So both the previous evidence together with our data suggest that SMARCA5 and CTCF can interact on DNA in AML blasts also within the Cohesin complex.

We show that Smarca5 is required for Ctcf association with chromatin at the ICR (Figure 1). Interestingly, this is not mutual - Smarca5 associates with chromatin independently of Ctcf. Other work has shown that CTCF interacts with the chromatin remodeling enzyme CHD8 (Chromodomain, helicase, DNA- binding protein 8), however, it is not required for CTCF binding to the ICR. Instead, CHD8 is required for CTCF-mediated enhancer-blocking activity at ICR H19, similar to Smarca5 [17].

What is the function of Ctcf/Smarca5 on DNA? The roles of both Ctcf and Smarca5 are complex as Ctcf and Smarca5 interact independently with other proteins. SMARCA5 interacts with protein complexes associated with nucleosome assembly and sliding: ACF [23] (with Acf1, a homologue of WCRF180), RSF [24] [25] [26], WICH (with Wstf) [27], NoRC [28], and NURF [29]. These reports also indicate that the binding partner of Smarca5 in each chromatin remodeling complex dictates the functional outcome. For example, the NoRC [28] complex has a transcriptional repressive effect while NURF is involved in transcriptional activation [29]. Our data indicate that Ctcf directs the effects of Smarca5. At the ICR locus, the Ctcf and Smarca5 cooperate in transcriptional regulation and thus inhibition of either Ctcf or Smarca5 lead to similar transcriptional outcomes (Figure 2). Upon perturbation experiments coupled by reporter assays (to determine enhancer-blocking effect) the Smarca5 knockdown appeared to be smaller compared to CTCF knockdown, however this can be attributed to higher stability of Smarca5 protein or differences in kinetics and level of knockdown. Nevertheless, Smarca5 with CTCF co-stimulates H19 expression and co-inhibits Igf2 expression (Figure 3). This suggests that Smarca5 supports Ctcf function as an enhancer-blocking co-factor at the ICR.

Our work further demonstrates an important role for CTCF/SMARCA5 in the regulation of *SPI1* gene expression in AML. Levels of PU.1 dictate differentiation outcome [22] and if *SPI1* transcription is downregulated by deletion of the URE it results in AML [13]. Our data show that CTCF, SMARCA5, and cohesin members are recruited to the *SPI1* gene (Figures 4 & 5) during AZA-induced myeloid differentiation. The Cohesin complex partially overlaps with CTCF/SMARCA5 occupancy on DNA indicating it contributes to their function, possibly in a similar fashion as at the ICR [2]. In AML progenitors, the *SPI1* gene is methylated and only SMARCA5 (without CTCF) can be detected at the URE while not at other regions. Upon DNA demethylation of AML blasts, the CTCF/SMARCA5 can newly interact with the −14.4 kb enhancer in the absence of cohesin (Figures 4 & 5), CTCF/SMARCA5 occupancy with cohesin is also restored at the −11 kb element. At the URE, CTCF occupancy is not recruited upon AZA while SMARCA5 and cohesin are detectable equivalent to normal differentiating myeloid cells (Figures 4 & 5). It remains not well understood how CTCF interaction with the −14.4 kb Enhancer upon AZA influences *SPI1* gene transcription but we speculate that it may not be sufficient to facilitate interaction of the URE with the promoter and additional enhancers leading to its upregulation [14]. Instead it is likely that CTCF and SMARCA5 interact with the −14.4 Enhancer and block *SPI1* transcription by employing the enhancer-blocking effects, similar to ICR. In addition, the DNA methylation pattern at the *SPI1* gene in AML likely disables binding of CTCF to the URE and other regions, leading to disruption of a cascade of transcriptional mechanisms possibly also involving looping of the URE and maybe other elements (at −11 kb) with the promoter.

What may be the mechanism of enhancer-blocking at *SPI1* gene used for? At the moment we do not have clear view but speculate that inhibition of PU.1 level is as important as its activation and may be used in blocking early differentiation decisions upon preceding DNA demethylation [30]. In addition, PU.1 silencing is of critical importance in erythroid lineage differentiation [31]. Alternatively, binding of CTCF/SMARCA5 at −14.4 Enhancer is aberrant and specific for AML undergoing DNA demethylation and may not exist in normal progenitor physiology. Nevertheless, while mechanisms of *SPI1* gene activation were previously described [14] this is one of the first reports of its transcriptional repression.

**Figure 8:**
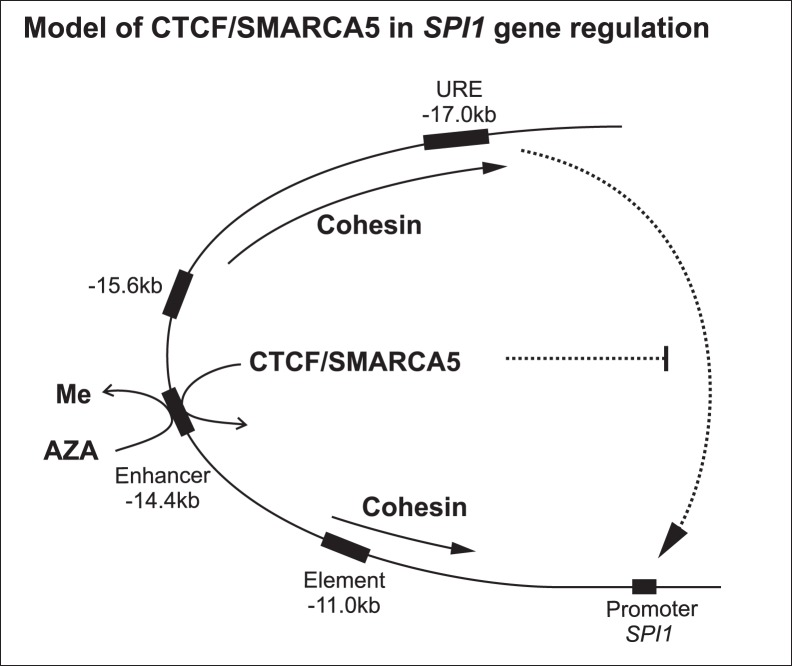
Model of epigenetic regulation of *SPI1* gene by CTCF and SMARCA5. CTCF binding site (−14.4 kb) becomes occupied by CTCF and SMARCA5 upon AZA-mediated DNA demethylation in AML blasts. Cohesin member’s recruitment partially overlaps with CTCF/SMARCA5 and display spreading over −11.0 kb and URE of *SPI1*. More diffuse occupancy of both CTCF and SMARCA5 at *SPI1* gene that was observed in mixed myeloid cells was not achieved in AML blasts, however the AZA treatment partially restored CTCF/SMARCA5 occupancy. Nevertheless, SMARCA5/CTCF is unable to potentiate *SPI1* derepression in AML blasts and instead, inhibits *SPI1* transcription possibly through the enhancer-blocking effect at the −14.4 Enhancer.

We herein present a model that involves the DNA methylation-sensing factor CTCF that cooperates on DNA with chromatin remodeling factor SMARCA5. At both studied DNA regions, ICR *H19* and *SPI1* gene regions, these factors co-occupy and co-regulate these targets in a cooperative fashion. While DNA hypermethylation in AML prevents binding of CTCF (and its partners) leading to structural properties characterized by repression of the *SPI1* gene, according to our ChIP data CTCF become recruited upon AZA in the −14.4 Enhancer while other regions were negative (Figure 4). This corresponds to DNA demethylation pattern of the −14.4 Enhancer (Figure S6). In addition, we think that also Smarca5 plays crucial role, as chromatin remodeling factor, for Ctcf recruitment on DNA (Figure 1C). Furthermore, members of Cohesin complex colocalize with CTCF and SMARCA5 at the URE and also at the −11 kb Element and presumably influence *SPI1* gene structure in differentiating myeloid cells. Demethylation therapy in AML is however not sufficient to restore *SPI1* gene transcription to the level found in cells undergoing normal differentiation (Figure 8).

## Supporting Information

Figure S1 mRNA expression of H19 and Igf2 genes in MEL cells. Expression is shown by Ct values (left). Amplification plot (right) is defined by X-axis with number of cycles and Y-axis with fluorescence signal (linear delta Rn).
(EPS)

Figure S2 Downregulation of Smarca5 expression in induced MEL-shSmarca5 cells. MEL- Smarca5 cells were treated with doxycycline (1 µM/ml) for 96 hours. Total mRNA was analyzed in 24 hr intervals (X-axis). Level of mRNA was determined by RT-PCR. mRNA level was normalized to Hprt1 and is shown as fold change relative to untreated cells (Y-axis), standard error (SE).
(EPS)

Figure S3 Co-IP of SMARCA5 and CTCF in K562 cells. Antibody for IP: Ctcf (upper panel) or Smarca5 (lower panel), Western blot for Smarca5 (upper panel) or Ctcf (lower panel). Arrows indicate specific signals.
(EPS)

Figure S4 Distribution of Ctcf and Smarca5 immunostaining in MEL cells (confocal laser scanning microscopy). MEL cells were fixed with 3,5% paraformaldehyde and immunostained with antibodies against Ctcf (green), Smarca5 (red), and DAPI (blue). Merge image as well as intensity profile graphs correspond to the section depicted by the line (see Merge). Fluorescence intensity profiles acquisition and determination of heterochromatin content was performed using ImageJ 1,38x software. Y-axis in intensity profile graphs represents fluorescence of respective fluorophor and X-axis represents distance.
(EPS)

Figure S5 mRNA expression of PU.1 and its target genes in OCI-M2 and normally differentiating mixed myeloid cells (heat map). mRNA from mixed myeloid cells, untreated OCI-M2 and OCI-M2 treated for 72 hrs with GCSF-AZA (see MM) were analyzed by RT-PCR. Y-axis: specific mRNA relative to average of HPRT1 and GAPDH was normalized to untreated OCI-M2.
(EPS)

Figure S6 **A** AZA-mediated DNA demethylation in untreated OCI-M2 and AZA-treated OCI-M2. Sequencing of bisulphite-treated DNA was performed over the regions −15.6 kb, −14.4 kb and −11.0 kb of *SPI1* locus. The primer sequences are shown in Table S2. Each region contains twelve CpGs as indicated below the graphs. **B** AZA-associated DNA demethylation was performed in CD34+ cells isolated from MDS patient without AZA therapy (N = 1, information in Table S1) and MDS patient after AZA therapy (N = 1). **C** DNA methylation of normal and AML/MDS CD34+ cells. The DNA was isolated from CD34+ cells of AML/MDS patients (N = 3) and control donor (N = 1). Y-axis: % of unmethylated CpGs; x-axis: number of CpG. Arrows indicate demethylated CpGs.
(EPS)

Table S1 Summary of patients.
(DOCX)

Table S2 Primer sequences.
(DOCX)
