# Correction: Epigenetic Control of *SPI1* Gene by CTCF and ISWI ATPase SMARCA5

**DOI:** 10.1371/journal.pone.0092635

**Published:** 2014-03-11

**Authors:** The PLOS ONE Staff

Figure 5 contains two extraneous graphs on the right-hand side. In addition, the error bars in Figures 4 and 5 are of inconsistent thickness. The authors have provided corrected versions here.

**Figure 4:**
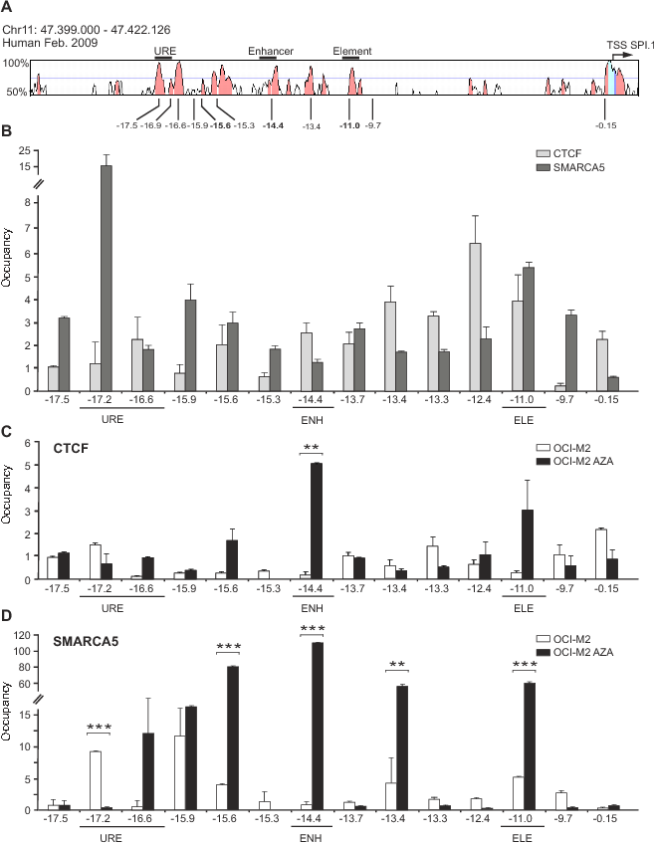
CTCF/SMARCA5 are recruited to *SPI1* locus in myeloid cells and upon AZA treatment in AML. **A:** Sequence conservation of human *SPI1* locus (VISTA) generated by aligning with murine DNA. Regulatory regions and positions of ChIP amplicons are numbered in respect to human *SPI1* TSS. **B:** ChIP of CTCF and SMARCA5 in mixed myeloid cells. **C:** ChIP of CTCF and **D:** SMARCA5 in OCI-M2 without (OCI-M2) or with AZA (OCI-M2 AZA) treatment. Y-axis: ChIP enrichment. X-axis: amplicons (distance relative to *SPI1* TSS). URE, Upstream Regulatory Element of *SPI1* gene; ENH, enhancer; ELE, element. Error bars: the standard errors (SE). Asterisks: p-values (t-test, 0.05–0.005).

**Figure 5:**
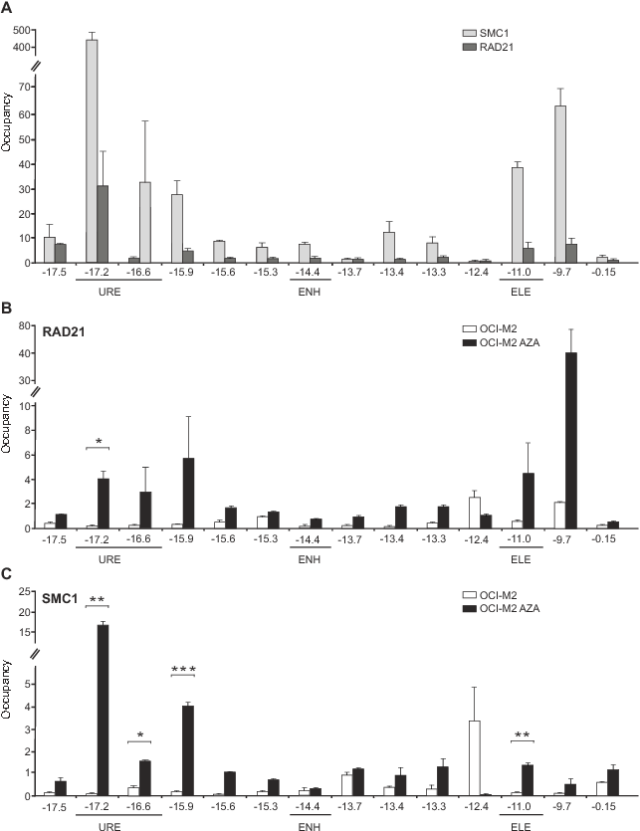
Binding of Cohesin complex members to *SPI1* locus. **A:** ChIP of RAD21 and SMC1 in mixed myeloid cells. **B:** ChIP of RAD21 and **C:** SMC1 in OCI-M2 without (OCI-M2) or with AZA (OCI-M2 AZA) treatment. Y-axis: ChIP enrichment. X-axis: amplicons (distance relative to *SPI1* TSS). URE, Upstream Regulatory Element of *SPI1* gene; ENH, enhancer; ELE, element. Error bars: the standard errors (SE). Asterisks: p-values (t-test, 0.05–0.005).
