# Supplementary material for: Epigenetic Control of SPI1 Gene by CTCF and ISWI ATPase SMARCA5
Source: PLoS One. 2014 Feb 3;9(2):e87448. doi: 10.1371/journal.pone.0087448 (PMC3911986; doi:10.1371/journal.pone.0087448)
Supplement: Table S1 — Summary of patients. (DOCX) [file pone.0087448.s007.docx]

**Table S1**

**AML**

**Summary of Patients (N=2) / Summary of Samples (N=2)**

| ***Clinical Parameters*** | ***Number of patients (N= )*** |
| --- | --- |
| **1. Age** |  |
| **(years)** | **V 282 (62), V361 (69)** |
| **2. Gender** |  |
| Male | **V361** |
| Female | **V282** |
| **3. Diagnosis (WHO Classification 2008)** |  |
| **AML with recurrent genetic abnormalities** |  |
| AML with t(8;21)(q22;q22); RUNX1-RUNX1T1 |  |
| AML with inv(16)(p13.1q22) or t(16;16)(p13.1;q22); CBFB-MYH11 |  |
| Acute promyelocytic leukemia with t(15;17)(q22;q12); PML-RARA |  |
| AML with t(9 ;11)(p22;q23); MLLT3-MLL |  |
| AML with t(6;9)(p23;q34); DEK-NUP214 |  |
| AML with inv(3)(q21q26.2) or **t(3;3)**(q21;q26.2); RPN1-EVI1 | **V 282** |
| AML with mutated NPM1* |  |
| AML with mutated CEBPA* |  |
| **AML with myelodysplasia-related changes** | **V361** |
| **Therapy-related myeloid neoplasms** |  |
| **Myeloid sarcoma** |  |
| **Myeloid proliferations related to Down syndrome** |  |
| **Blastic plasmacytoid dendritic cell neoplasm** |  |
| **4. Cytogenetic** (Risk Category) |  |
| Favorable (t(8;21), t(15;17), inv(16)) |  |
| Intermediate (Normal, +8, +21, +22, del(7q), del(9q), Abnormal 11q23, all other) | **V282, V361** |
| Unfavorable (-5, -7, del(5q), Abnormal 3q, Complex cytogenetics) |  |
| **5. Overall Survival (From Diagnosis to last follow up)** |  |
| <6 months |  |
| 6 - 24 months | **V282** |
| >24 months |  |
| **6. Therapy** |  |
| Sample before treatment | **V282, V361** |
| BSC (best supportive care) |  |
| Standard induction chemotherapy (7+3) |  |
| Demethylation drug Azacitidine (Vidaza) |  |
| Other |  |
| **7. Response (IWG Criteria)** |  |
| Non-response |  |
| HI (Hematologic Improvement) |  |
| PR (Partial remission) | **V361 (after 4. AZA)** |
| CR (Complete remission) |  |
| CRi (incomplete blood count recovery) |  |
| PD (disease progression) | **V282** |
| Relapse |  |
| **8. Time of the study (during)** |  |
| \| date of start \| date of end \| \| --- \| --- \| | \| **19. 4. 2010** \| **30. 5. 2013** \| \| --- \| --- \| |
| Time of study (observation) in months |  |
| **9. Outcome** |  |
| Died (during the study) | **V282 (31. 3. 2011),**  **V361 (19. 3. 2012)** |
| Alive (after study) |  |

**MDS**

**Summary of Patients (N=2) / Summary of Samples (N=2)**

| ***Clinical Parameters*** | ***Number of patients (N= )*** |
| --- | --- |
| **1. Age** |  |
| **(years)** | **V285 (67), V367(69)** |
| **2. Gender** |  |
| Male | **V367, V285** |
| Female |  |
| **3. Diagnosis (WHO 2008)** |  |
| RA (Refractory cytopenia) |  |
| RARS (Refractory anemia with ring sideroblasts) |  |
| RARS-t (Refractory anemia with ring sideroblasts – thrombocytosis) |  |
| RCMD (Refractory cytopenia with multilineage dysplasia) |  |
| RAEB-I (Refractory anemia with excess blasts 5-9% blasts) | **V285** |
| RAEB-II (Refractory anemia with excess blasts 10-19% blasts) | **V367** |
| CMML (Myelodysplastic-myeloproliferative overlap syndromes) |  |
| 5q- syndrome |  |
| Myelodysplasia unclassifiable |  |
| **4. Cytogenetic** (IPSS) |  |
| Favorable (Normal, 5q−, −Y, 20q−) | **V367 (del 20), V285 (del20)** |
| Intermediate (All others) |  |
| Unfavorable (Complex, −7/7q−) |  |
| **5. IPSS** |  |
| Low risk |  |
| Int I |  |
| Int II | **V367, V285** |
| High risk |  |
| **6. Overall Survival (From Dg to LF)** |  |
| <12 months |  |
| 12-36 months |  |
| >36 months | **V367, V285** |
| **7. Therapy** |  |
| Sample before treatment | **V367** |
| Azacitidine (Vidaza) | **V285** |
| Other (chemotherapy) |  |
| BSC (best supportive care) |  |
| **8. Therapy response** (IWG criteria 2006) |  |
| Non-response |  |
| HI (Hematologic Improvement) |  |
| PR (Partial remission) | **V285** |
| CR (Complete remission) |  |
| Relapse |  |
| Transformation to AML |  |
| **9. Time of the study (during)** |  |
| \| date of start \| date of end \| \| --- \| --- \| | \| **19. 4. 2010** \| **30. 5. 2013** \| \| --- \| --- \| |
| Time of study (observation) in months |  |
| **10. Outcome** |  |
| Died (during the study) | **V367 (5. 12. 2010)** |
| Alive (after study) | **V285** |
