# Supplementary material for: Epigenetic Control of SPI1 Gene by CTCF and ISWI ATPase SMARCA5
Source: PLoS One. 2014 Feb 3;9(2):e87448. doi: 10.1371/journal.pone.0087448 (PMC3911986; doi:10.1371/journal.pone.0087448)
Supplement: Table S2 — Primer sequences. (DOCX) [file pone.0087448.s008.docx]

**Table S2**

**RT-PCR**

| Primer | Sequence |
| --- | --- |
| mGapdh(mRNA)-F#77 | ACTTTGTCAAGCTCATTTCCTGGTATG |
| mGapdh(mRNA)-R#77 | TTTCTTACTCCTTGGAGGCCATGTAG |
| mHprt1(mRNA)-F#95 | CTTCCTCCTCAGACCGCTTTTTGCC |
| mHprt1(mRNA)-R#95 | TAACCTGGTTCATCATCGCTAATCACG |
| mSnf2h(mRNA)WM-F#88 | TGCTTTCAGTTGGAGATTACCGACACC |
| mSnf2h(mRNA)W-R1#88 | TGATGGAGAATCTTCAAATCGAGTACAAAC |
| mIgf2(mRNA)-F2#40 | ACGCTTCAGTTTGTCTGTTCGGACC |
| mIgf2(mRNA)-R2#40 | AAGCAGCACTCTTCCACGATGCCAC |
| mH19(mRNA)-F#67 | GTCTCGAAGAGCTCGGACTG |
| mH19(mRNA)-R#67 | ACTGGCAGGCACATCCAC |
| mSfpi1(mRNA)-F#94 | CTGATGGAGAAGCTGATGGCTTGG |
| mSfpi1(mRNA)-R#94 | AGCAGGAACTGGTACAGGCGAATC |
| mCebpa(mRNA)-F#67 | AAACAACGCAACGTGGAGA |
| mCebpa(mRNA)-R#67 | GCGGTCATTGTCACTGGTC |
| hGAPDH #60 F | AGCCACATCGCTCAGACAC |
| hGAPDH #60 R | GCCCAATACGACCAAATCC |
| hHPRT #73 F | TGACCTTGATTTATTTTGCATACC |
| hHPRT #73 R | CGAGCAAGACGTTCAGTCCT |
| hSPI1 #27 F | CCACTGGAGGTGTCTGACG |
| hSPI1 #27 R | CTGGTACAGGCGGATCTTCT |
| hCEBPA F #28 | GGAGCTGAGATCCCGACA |
| hCEBPA R #28 | TTCTAAGGACAGGCGTGGAG |
| hITGAM(mRNA)-F#36 | ACCCCTGGTTCACCTCCT |
| hITGAM(mRNA)-R#36 | CATGACATAAGGTCAAGGCTGT |
| hMPO(mRNA)-F#38 | CGTCAACTGCGAGACCAG |
| hMPO(mRNA)-R#38 | GTCATTGGGCGGGATCTT |
| hGelBmRNAp6F | GAACCAATCTCACCGAGG |
| hGelBmRNAp6R | GCCACCCGAGTGTAACCATA |
| hCSF1R F #14 | TCTGGTCCTATGGCATCCTC |
| hCSF1R R #14 | TGCCAGGGTAGGGATTCA |
| hCSF3R F #18 | GTCCAAGATCACAAAGCTGGT |
| hCSF3R R #18 | CCGCACTCCTCCAGACTTC |
| hFOS F #67 | CTACCACTCACCCGCAGACT |
| hFOS R #67 | AGGTCCGTGCAGAAGTCCT |
| hEGR2(mRNA)-F#3 | AACGGAGTGGCCGGAGATGGCATG |
| hEGR2(mRNA)-R#3 | TGCAGAGACGGGAGCAAAGCTGCTG |
| hCTCF (mRNA)-F#55 | CCAGAGCCTCAGCCTGTGACCC |
| hCTCF (mRNA)-R#55 | CAACCTGAATGATAGCTGTTGGCTGG |
| hSMARCA5(mRNA)-F#32 | AGTAACCAACAGTGGCAAAATGGTG |
| hSMARCA5(mRNA)-R#32 | TGACTGAAGATTAGTACTCGTGAACC |

**ChIP**

| Primer | Sequence |
| --- | --- |
| mH19(-2.2kb)-F | GTTCACTCTCCACGCTGTGCAG |
| mH19(-2.2kb)-R | TCACAGCGGACCCCAACCTATG |
| mH19(-2.6kb)-F | CAAAACCAGCCAGTGTGGCTCAC |
| mH19(-2.6kb)-R | AGTTGGCAGCATTTGGGCCACG |
| mH19(-3.1kb)-F | TGGCTGGTTTGTGGCAGATAATCG |
| mH19(-3.1kb)-R | GCATAGGTGTCCTGCCTTCTGC |
| mH19(-3.7kb)-F | CAACAAGGTCGGCTTACTCTCTGC |
| mH19(-3.7kb)-R | CCGTTTTAGGACTGCGATGTACGAG |
| mH19(-3.9kb)-F | TGCTACATTCACACGAGCATCCAGG |
| mH19(-3.9kb)-R | GGCTCTTTAGGTTTGGCGCAATCG |
| hPU.1 (-17.5kb)-F | GGATGGCTGAGGTTGATGGTTGA |
| hPU.1 (-17.5kb)-R | CAGCAGACAGGGATGAAGACAGAAGA |
| hPU.1 (-17.2kb)-F | CCGCACTCCTCCAGACTTC |
| hPU.1 (-17.2kb)-R | CATGGGGACCTGCCAGGCAG |
| hPU.1 -16,6 F | CCTGACCCCACATTCTGATT |
| hPU.1 -16,6 R | CTTCTTCTGGGCTCTCAGC |
| hPU.1 -15,62 F | ATCTGGGAGGACTAGGCTTT |
| hPU.1 -15,62 R | AATGACCGCGTCTTCTTTCT |
| hPU.1 -15,28 F | GCTTCTGGTCAAGTTTGCTG |
| hPU.1 -15,28 R | GGACTTGGGTTTTAGGGGAG |
| hPU.1 -14,36 F | TCATGCTTGGAAACACTCCA |
| hPU.1 -14,36 R | TGTCTGCTTGAAGGTCAAGG |
| hPU.1 (-13,7kb)-F | GGCAGGAAGATCACTTGAGCCCAG |
| hPU.1 (-13,7kb)-R | GCAATCCTCCCACCTCAGCTTCC |
| hPU.1 (-13.4kb)-F | TGTGACCAGGGCAGGAGCAGG |
| hPU.1 (-13.4kb)-R | GCCAGCCAGGGAAGGTAACAGG |
| hPU.1 (-13,3kb)-F | CCTCCTGACCAGTCCCAACTGAGG |
| hPU.1 (-13,3kb)-R | TGTAGAAACAGCTTGTGCCCAGCAC |
| hPU.1 (-12,47kb)-F | GGGCATCCCTGCACTGTTCTTG |
| hPU.1 (-12,47kb)-R | CTGAGTGTGTGCCTTGAGCCCTTG |
| hPU.1 (-11.0kb)-F | TGGCAGCCTGGAGGAGGCAAG |
| hPU.1 (-11.0kb)-R | GGACACCGCTCAGCATTCCTGG |
| hPU.1 (-9.7kb)-F | AGCCTCTCCCAGTGTCTCAACCAT |
| hPU.1 (-9.7kb)-R | TAGCAACCTGTCCCCATAGGGTGT |
| hPU.1 (-0,15kb)-F | TCCCTCTCAGTCCCAGCTTCCTC |
| hPU.1 (-0,15kb)-R | CCGTTTGCATAAATCTCTTGCGCTAC |

**DNA Methylation**

| Primer | Sequence |
| --- | --- |
| PU.1-URE-F | GAGAAATGGTTTTTTTGTGATTT |
| PU.1-URE-R | ACAACTACCCCTATTTCCACAT |
| hPU.1 (-11kb)-F conv. | GGTTAGTAGGGATAAGGGTGTG |
| hPU.1 (-11kb)-R conv. | TCAAAACCCCTCAAACCTATAC |
| hPU.1 (-14,4kb)-F3 conv. | AAATTAGGAGAAGTGAAGTGGTTG |
| hPU.1 (-14,4kb)-R3 conv. | CCATCTATCCCAACTCAAATCTATA |
| hPU.1 (-15,6kb)-F conv. | GTGGGAGGAGTAGGTTTATGTT |
| hPU.1 (-15,6kb)-R3 conv. | CCTCCCCTTCTTACAATATCAAC |
